# Co-Design of a Health Screening Program Fact Sheet by People Experiencing Homelessness and ChatGPT: Focus Group Study

**DOI:** 10.2196/68316

**Published:** 2025-07-04

**Authors:** Nóra Radó, Orsolya Németh, Sándor Békási

**Affiliations:** 1 DocRoom Health Research Program, Hungarian Charity Service of the Order of Malta, 28 Bem Rakpart, Budapest, H-1011, Hungary, 36 12018517; 2 Institute of Behavioural Sciences, Faculty of Medicine, Semmelweis University, Budapest, Hungary; 3 Department of Community Dentistry, Faculty of Dentistry, Semmelweis University, Budapest, Hungary; 4 Centre for Translational Medicine, Semmelweis University, Budapest, Hungary

**Keywords:** digital health, co-design, oral health, focus group, expert by experience, ChatGPT, health information, leaflet, screening, mixed method study, screening programs, oral cancer, vulnerable population, generative AI, co-design, Hungary, homeless, qualitative method, quantitative method, artificial intelligence

## Abstract

**Background:**

People experiencing homelessness have worse oral health outcomes and a notable health informational asymmetry compared to the general population. Screening programs present a viable option for this population; however, barriers to access, such as lower levels of health literacy, lack of information, and mistrust, narrow their chances to participate in such programs.

**Objective:**

The aim of this study is to investigate the applicability of generative artificial intelligence (AI) in designing a homeless health screening program fact sheet with experts by experience using co-design principles.

**Methods:**

Six fact sheet text variants were created by the open-access version of ChatGPT 3.5 for an oral cancer screening program targeting people experiencing homelessness in Budapest, Hungary. Clients of homeless social services (N=23) were invited to a short questionnaire survey and 3 semistructured focus group discussions between May and July 2024. General opinions regarding generative AI technology and direct feedback on the text variants were obtained. Additionally, a standardized readability assessment of the text variants was completed via the Sydney Health Literacy Lab Editor.

**Results:**

Almost two-thirds of participants (17/23) stated that they had previously heard about AI; however, their self-assessment regarding the extent of their knowledge resulted in an average of 2.38 (n=16) on a 5-point Likert scale. During the first focus group discussion, all 6 variants received a high score (between 4.63 and 4.92 on a 5-point Likert scale). In the next sessions, participants remained positive when the pool was narrowed to 4 versions, although they scored the texts lower. During open discussions, text variants were considered understandable, while difficulties with medical expressions, lengthiness of sentences, and references to a stereotypical homeless subgroup (rough sleepers) were also reported. The health literacy editor showed that most AI-generated text variants were difficult to read and too complex for the target group.

**Conclusions:**

The co-design process revealed that focus group participants actively wanted to shape the fact sheet drafts. They shared their insights on how to make the text variants more appealing for the target audience. Moreover, the involvement of generative AI technology revealed that the participants have heard about the concept of AI and text generation as a potential function, and they have not rejected its use in health care settings.

## Introduction

### Homelessness and Oral Health

Homelessness is a complex social phenomenon that leaves individuals for a shorter or longer period in an extremely vulnerable life situation. According to previous research, homelessness is associated with significantly higher disease burden [1-3] and higher mortality rates for both women and men than the average population [4]. In Western, high-income countries, studies have also shown that homelessness is an independent risk factor for mortality, and life expectancy varies between 50‐65 years on average [5].

Previous research on the oral health of people experiencing homelessness found that this population has poor outcomes; they are in great need of restorative, oral hygiene, and periodontal treatment. They also have inadequate access to dental services, mostly relying on emergency treatment, in parallel with unmet treatment needs [6-8]. In the United Kingdom, dental health was identified as this group’s largest unmet health need [9]. In the United States, a national study of homeless adults using Health Care for the Homeless services found that approximately half of homeless adults had an unmet need for dental care [10]. Higher rates of substance use (alcohol, tobacco, drugs) further put the oral and general health of people experiencing homelessness at risk [11]. Freitas et al [10] found strong associations between having lost half or more of their teeth and evidence of problem drinking, cocaine use, or having ever smoked. In 1997, in Hungary, precancerous lesions were found in or around the oral cavity in 14% of people experiencing homelessness or participating in alcohol withdrawal treatment, benign tumors in 2.33%, and malignancies in 2.66% [12].

Access to oral care also comes with serious barriers for this population; the cost of care for private service providers, lengthy waiting lists for publicly funded institutions, competing priorities (which might lead them to secure food and accommodation before health care), a lack of information, mistrust of health care systems, and experiences of discrimination in care settings all drive people experiencing homelessness away from dental care services, resulting in them needing to rely on emergency treatment in cases of acute problems [11,13-15]. Moreover, psychosocial factors play a significant role; higher levels of dental anxiety and dental phobia were found in the homeless adult population [16].

### Screening Programs, Health Literacy, and Information Asymmetry

As literature shows, the potential implications of a health screening program in dental practice are reductions in morbidity, mortality, and onward cost to health care systems by avoiding acute presentations of late-stage chronic diseases [17]. Moreover, Nunez et al [18] found that in the United States, veterans who received dental care were found to stay in homeless intervention programs significantly longer than veterans who did not. Their findings also indicated that the impact of the provision of dental care on outcomes among homeless veterans is equivalent to the impact of psychological treatments for depression.

To overcome the barriers to dental care for people experiencing homelessness in Hungary, the Charity Service of the Order of Malta, in collaboration with Semmelweis University and Óbuda University, launched an oral cancer screening program with digital capabilities in Budapest in 2024. The initiative fits into the wider digital health research agenda of the Charity Service, which previously completed numerous digital health projects [14,19-21]. Using advanced asynchronous telecare solutions in this vulnerable community, the new digital platform Lesionwizard was designed to deliver an oral cancer screening program for people experiencing homelessness using teledentistry [22].

As an additional barrier, a lack of information seriously burdens vulnerable populations. One of the main problems is information asymmetry between providers and people experiencing homelessness, coupled with lower levels of (oral) health literacy. In our previous study in collaboration with the Digital Health Working Group at Semmelweis University, Budapest, Hungary, we found that difficulties in gaining reliable information from service providers might result in the phenomenon that people experiencing homelessness look up medical information online or turn to alternative sources [14]. Csikar et al [23] also identified the level of (oral) health literacy as a barrier for people experiencing homelessness who had difficulties understanding letters sent to them. The authors concluded that it impacted their prioritization of oral health, as individuals may have yet to understand the importance of oral care or their options for accessing it.

### The Application of Co-Design and Generative Artificial Intelligence

To facilitate participation in our oral cancer screening program, the research team decided to aid the initiative with an A5-format, awareness-raising, short health information fact sheet that presents the initiative as acceptable, available, and effective for this vulnerable population [13]. Co-design principles and the technological assistance of the generative artificial intelligence (AI) tool ChatGPT (OpenAI) were applied.

Co-design has previously been defined as a participatory approach that brings individuals together to collaborate and combine their knowledge, skills, and resources to accomplish a design task [24], also in the area of digital health for tool, educational, and health information material development [24-28]. It involves the meaningful engagement of end users recognized as experts by experience [29]. Previous research found that co-design, co-creation, or co-production can be empowering for socially marginalized or excluded groups, such as people experiencing homelessness, while it is also a pivotal approach to tackling stigmatization and promoting inclusivity. Co-design techniques resulted in increased applicability and acceptance of research questions, outputs, participant engagement, and knowledge of different contexts, as well as an improved community network for the researchers [30].

Generative AI software, such as ChatGPT, is a large language model (LLM) combined with a user-friendly interface that uses deep learning algorithms trained on vast amounts of data to generate multimodal humanlike responses to user prompts [31]. Its applicability in medicine is currently under scrutiny, but it has great promise in aiding doctor-patient communication and providing patient information. It has performed satisfactorily in answering physician-generated medical queries across 12 distinct specialties [32]. It has also been shown to simplify online health information [33], to generate dermatologic patient education materials according to specific reading levels [34], and to translate patient education materials from English into other languages [35].

In this research project, we aimed to co-design an awareness-raising fact sheet for an oral cancer screening program with people experiencing homelessness as experts by experience and ChatGPT. The latter was used to present textual alternatives for this health information piece, so we could also test the usability of ChatGPT in designing adequate information materials serving the needs of people experiencing homelessness.

## Methods

### Participants and Recruitment Procedure

The study followed the Consolidated Criteria for Reporting Qualitative Research (COREQ) checklist, adapted to focus groups [34] (see Checklist 1). Three focus group discussions were organized to provide feedback regarding patient information materials for an oral cancer screening program. One of them was an already existing group of experts by experience; in addition, two ad hoc groups were formed from clients of 3 shelters in Budapest, Hungary (Miklós Street Integrated Homeless Care Center, Homeless Care Center at Bem rakpart, and Galvani Street Homeless Care Center), operated by the Hungarian Charity Service of the Order of Malta. The sample constituted a convenience sample; the researchers advertised ad hoc focus groups in the shelters, and clients over 18 years without mental health problems and dementia who expressed their interest participated voluntarily, without any compensation.

The experts by experience group was established in 2023 to assist in co-designing initiatives targeting relevant health issues of people experiencing homelessness. Expert group meetings were organized on a monthly schedule with the attendance of 6‐9 experts. The option to participate in the experts by experience group was open to adult clients (>18 years) of homeless shelters operated by the Hungarian Charity Service of the Order of Malta, without mental health problems or dementia.

From the recruited sample (N=26), three people decided not to participate (2 people due to scheduling problems and 1 person due to the difficulty of the topic). The number of participants in the 3 focus groups was 6, 10, and 7, and the demographic characteristics are shown in Table 1. The focus group discussions took place on May 16, June 4, and July 4, 2024; their length varied between 40 and 55 minutes.

**Table 1.** Demographic characteristics of the focus groups discussing ChatGPT-generated text variants. Participants were clients of 3 homeless shelters operated by the Hungarian Charity Service of the Order of Malta.

Group	Age (years), mean (SD)		Gender, female (male)	
Experts by experience (n=6)	55.83 (14.97)		1 (5)	
Focus group 2 (n=10)	61.50 (7.11)		2 (8)	
Focus group 3 (n=7)	53.57 (5.19)		0 (7)	

### Text Generation

Six text variants of basic client information materials were generated on May 13, 2024, by the open-access version of ChatGPT 3.5 developed by OpenAI. The researchers chose OpenAI’s most advanced freely available product because, according to statistics, it is the most widely available [36]. Prompts were applied in English, while the results were given in Hungarian. Each text version was limited to a word count of 150 due to the limitations of an A5-size one-sided fact sheet. All prompts emphasized the target population (people experiencing homelessness), the main aim of the text (to raise the level of participation), and a reasoning or style/tonal requirement. These requirements were the following: (1) scientific evidence regarding oral cancer, (2) statistical evidence regarding oral cancer, (3) as motivating as possible, (4) based on an informal, familiar tone, using slang expressions, (5) formatted as a clickbait news article, and (6) structured in a bullet-point format. Otherwise, the prompts were formulated as plain texts produced by people without relevant expertise in prompt design, as the researchers had the intention to involve ChatGPT as a tool that would be used by nonexpert social sector users. The prompts used in this study and the resulting Hungarian text variants, as well as the texts translated into English, are provided in Multimedia Appendix 1.

### Feedback Questionnaires and Semistructured Group Discussions

A 2-part short feedback questionnaire developed by the research team was used to quantify different aspects of AI in general and AI-generated text variants, and it was also used to catalyze an open group discussion. The first part consisted of three items: (1) whether the participants have heard about AI (in a Yes or No scheme), (2) self-assessment of knowledge regarding AI technology (on a 5-point Likert scale), (3) and trust in its use in health care settings (on a 5-point Likert scale). The second part included each text variant with 7 items. Assessment of understandability and clarity, the quality of information content, the tone and style of the texts, and the convincing factor were conducted on a 5-point Likert scale. Lastly, 3 open questions inquired about the strengths and weaknesses of the texts, any changes suggested, and the applicability of the texts in the screening program. The quantified values were obtained in paper and pencil form, while answers to open questions were discussed by the group members, and notes were taken by the research team.

### Text Evaluation via the Sydney Health Literacy Lab Health Literacy Editor

After the focus groups analyzed the text variants, we also assessed the texts according to standardized readability measurement tools. There are several methods to calculate the readability scores of texts, such as the Flesch-Kincaid method [37], the Gunning fog index [38], or the SMOG readability formula [39]. The third method is frequently used in health research [40]. In this study, we used the framework by Ayre et al [33] entitled the Sydney Health Literacy Lab (SheLL) Health Literacy Editor as it is a web-based tool designed to objectively assess the extent to which health information is written in plain language, while all the other methods serve as general tools for readability measurement. The SHeLL Editor, available as a web-based tool [41], assesses the number of words, readability as grade reading score, language complexity, passive voice usage, and the use of bullet points for lists [33]. Based on this framework, we made the first 4 assessments and left out bullet points for lists as they only appeared in 1 text variant. The text assessments were then compared with the focus group assessment.

### Ethical Considerations

Participation in the focus group discussion was voluntary and without any compensation. Data collection from the questionnaires was anonymous, and notes from the focus group discussions were deidentified. After a verbal summary of the study tasks and setting the ground rules of the focus groups, consent was obtained from all members of the group, and questionnaires were collected anonymously. During the focus group discussions, no dropout occurred. As an observational, noninterventional, and nonbiomedical investigation of the study subjects’ sociological behavior, it was exempt from ethical review, as it is out of the scope of the Hungarian Act CLIV of 1997 on Health Care, the Decree 23/2002 (9 May) of the Ministry of Health on Medical Research on Human Subjects, and the Decree 35/2005 (26 VIII) of the Ministry of Health on the Clinical Investigation of Investigational Medicinal Products for Human Use and the Application of Good Clinical Practice [42]. For the same reason, the Semmelweis University Committee for Regional Institutional Scientific and Research Ethics could not issue an institutional review board exemption.

## Results

### General Acceptance of AI

During the focus group discussions, participants were asked about AI technology as a starting point. Of the 23 participants, 17 (74%) stated they had heard about AI in a Yes or No scheme. On a 5-point Likert scale asking about the extent of their knowledge of AI, they were more hesitant, resulting in an average of 2.38 (n=16), where 1 was not familiar at all and 5 was totally familiar. As examples of the possible functions of AI, text or picture generation was mentioned the most (8 times), and in 3 cases, AI-generated content was attributed as “fake” or “not real.” One participant said:

I know it can also generate fake photos.

After a general impression of AI, its application in health care was also discussed. For the question “Would you trust in AI-generated medical texts, documents, or tools?” the answers averaged 3.06 (n=16) on a 5-point Likert scale (where 1 was no trust at all and 5 was complete trust). When participants were asked about the reasoning behind their answers, the need for human involvement was emphasized concerning decision-making regarding health issues. Two participants said the following:

Even if it was created by humans, machines can have errors, so I would have less confidence in it if my health were at stake.

I have no opposition regarding artificial intelligence if they use it as a helping tool, but it would be frightening for me if it were to make decisions without human oversight.

### Applicability of Text Variants

In the focus group discussions, the AI-generated text variants were presented. As the first step before using these texts, 2 independent researchers reviewed the AI-generated draft text variants. Modifications were applied in only 2 cases due to severe grammatical errors in the Hungarian language that limited the integrity of these texts. Otherwise, all variants were intact and brought to the focus groups in their original form. The source of each text was clarified for members of the groups only during the closure of group sessions.

First, participants were asked to provide general feedback on the applicability of each text variant in the context of a future oral cancer screening program. Scores measured on a 5-point Likert scale were detected in 4 dimensions (understandability and clarity, the quality of information content, the tone and style of the texts, and their convincing factor), and the results are shown as the average of these 4 items. During the first focus group discussion with experts by experience, all 6 variants were presented to the group.

Although the expert group members were highly positive regarding all variants, there were slight differences in the scoring of the text versions. The ranking turned out to be the following: (1) scientific reasoning (4.92; n=6), (2) informal, familiar tone (4.83; n=6), (3) focusing on motivation (4.75; n=6), (4) clickbait news article style (4.71; n=6), (5) statistical reasoning (4.67; n=6), and (6) bullet-point format (4.63; n=6). Participants were also asked to agree on the two most promising text variants that represented the highest opportunity to raise the attendance rate according to their experience. A consensus was reached after a short discussion, resulting in the variant based on scientific reasoning being selected as the top choice, and the informal, familiar version as the second choice, without knowing the quantitative results. Participants were convinced that different text variants could address different subgroups of people experiencing homelessness. One participant remarked the following:

The familiar one will motivate the youth more. It sounds not so official.

After the first focus group discussion, 2 text variants (number 2 with statistical reasoning and number 6 with a bullet-point format) were removed from the pool as these were highly redundant according to the previous participants, and going through 6 texts challenged their attention, limiting the effectiveness of group discussions. The remaining 4 variants were presented to both remaining focus groups in the same form.

Participants of the latter two group discussions (n=17) were more critical in all aspects of the quantitative survey. The results of the 5-point Likert scale scoring were the following: (1) informal, familiar tone (3.77; n=13), (2) focusing on motivation (3.69; n=15), (3) scientific reasoning (3.69; n=16), and (4) clickbait news article style (3.50; n=12).

### Evaluation of AI-Generated Content by Research Participants

After scoring all text versions, an open discussion took place. All group discussions concluded that the texts are almost fully understandable. Two participants remarked the following:

I can totally get what they are speaking about.

The main point is clear, even if there are difficult words.

However, there were suggestions for certain changes related to wording for ease of reading. The replacement of medical jargon—from “oral cancer” to “mouth cavity tumor,” as the latter is a more commonly used term by the general population in the Hungarian language—was mentioned 7 times and affected all variants, while words with Latin roots, for example, “informing” and “early staging,” were advised to be changed to a more widely used expression one time each.

In addition, the length of sentences as a factor causing gaps in readability was mentioned twice in the context of the versions based on scientific and statistical reasoning. Furthermore, participants accommodated in night shelters and other temporary housing solutions mentioned that the phrasing in two-thirds (4/6) of the text variants was not inclusive enough, as the term “rough sleepers” was used as a synonym for the homeless population, and this might result in the alienation of other subgroups. As one participant said:

They say people living on the streets only. That’s not very motivating for me, who is living in a shelter.

Based on the focus group discussions, the research group summarized the main strengths and weaknesses of the text variants created by ChatGPT 3.5 in Textbox 1.

Textbox 1. **Evaluation of strengths and weaknesses of the ChatGPT-generated health information content of 6 text variants by people experiencing homelessness.**

**Strengths**

- No significant opposition was detected against AI-created content from people experiencing homelessness.
- It is easy to generate many text outputs with open-access tools quickly.
- The results are almost ready to use, with minimal modification needed from the textual coherence point of view (in the Hungarian language).
- In most cases, participants were positive about whether the texts could fulfill the goal of motivating the target population to attend the program.
- Text variants in various tones and styles can attract different age groups.

**Weaknesses**

- There was a level of disapproval, mostly regarding AI-based decision-making processes concerning health issues.
- Text variants repeated the same problems (eg, medical jargon is difficult to understand for vulnerable populations).
- The motivational elements of text variants were stereotypical to a subgroup of people experiencing homelessness (rough sleepers) and lacking other prominent subgroups (eg, people accommodated in community shelters or temporary hostels).

### Assessment of Text Variants With the SHeLL Editor

The research participants mentioned during the focus group discussions that text variants presented words that were difficult for them to understand, so to have a more comprehensive understanding of the ChatGPT-generated text variants’ readability level, we evaluated the text with the help of the SHeLL Editor [33,38,43]. As this tool is only available in English, we translated the Hungarian text variants into English. The assessment of the text variants based on their word count, grade reading score, language complexity in percentages, and passive voice usage is summarized in Table 2.

**Table 2.** Evaluation of the readability of the 6 ChatGPT-generated text variants with the Sydney Health Literacy Lab Health Literacy Editor.

Text versions	Word count (sentence count)	Grade reading score	Text complexity, %	Passive voice, word count
Scientific evidence	87 (6)	11.5	26.6	0
Statistical evidence	81 (5)	10.4	21.4	0
Motivational	90 (7)	9.2	16.2	0
Informal	94 (11)	7.4	7.3	0
Clickbait news article	100 (12)	10.3	22.3	0
Bullet point format	65 (6)	8.3	21.2	0

Grade reading score refers to how difficult a text is to read and roughly corresponds to the expected reading ability for US school students in different grades [33]. Text complexity means the proportion of the text (%) that contains acronyms, uncommon words (as defined by an existing English-language corpus), or terms listed as public health or medical jargon [43].

## Discussion

### Main Findings

Our aim to co-design an awareness-raising fact sheet for an oral cancer screening program with people experiencing homelessness and ChatGPT was realized. We were also able to test the usability of ChatGPT in designing adequate information materials serving the needs of people experiencing homelessness by having focus group participants evaluate the ChatGPT-generated text variants. Moreover, focus group participants expressed prior knowledge of the concept of AI. Of potential functions of AI, they mentioned text or image generation the most. It also turned out that they did not reject the medical use of AI, although they indicated hesitancy in trusting it, especially without human oversight.

The text evaluation included cohesiveness, wording, tone, and style, and the results showed that, overall, the texts were able to fulfill their purpose of motivating the target group to participate in the screening activities, although participants suggested that the wording could be less stereotypical and less difficult to read. They also mentioned that text variants with different tones and styles could attract different age groups from the diverse population of people experiencing homelessness. The readability assessment of the texts underpinned their findings as the readability level of the majority of the text variants was above the readability level recommended for health-related texts by the literature [44].

### Applicability of Generative Software in Health Care

Many fields of possible applications have been raised in using generative AI in clinical settings, such as writing discharge summaries [44], medical notes based on transcripts of physician-patient encounters, summaries of laboratory test results [45], medical education [46], medical research [47], providing a communication platform for patients, and facilitating health information dissemination [47]. One of the most obvious applications is generating tailored patient information on a predetermined topic, as collecting massive amounts of available evidence on different topics and human-like reasoning are easily achievable with open-access versions of generative software.

However, vulnerable populations might have different contexts, motivations, challenges, and medical needs than the general population and often require tailored medical treatment approaches to ensure the safety and efficacy of the treatment alongside potentially optimal health outcomes [48]. Moreover, concerns have arisen that the quality of AI-generated results depends on the user’s ability to develop effective prompts, input accurate text for inquiries, and access advanced features through subscriptions; as a result, individuals with limited health literacy, insufficient prompt development skills, or an inability to afford premium subscriptions may miss out on these technological benefits, potentially exacerbating health disparities [49].

### Vulnerable Groups and Their Knowledge and Trust Around AI

In health care, underserved subgroups are known to have limited access to care pathways and possess altered demands in addition to an existing systematic information asymmetry, as our previous study also revealed [14]. As the results showed, anxiety, misunderstanding, discrimination, and negative experiences related to this information deficit could be compensated for by using co-design principles. Better usability of such services might play an important role in the more equitable management of health issues. Moreover, the usage of ChatGPT as a co-design element might unburden health care and social care personnel tasked with the formulation of client information, as creating relevant materials with appropriate prompts takes significantly less time than building them from scratch. On the other hand, editing the draft or iterating the prompt sequence may require a level of expertise and take additional time, making the time-saving element of the use of ChatGPT unclear. Further on, as another potential downside of relying on the technology, it is questionable whether and for how long the subscription-based model of OpenAI or any other generative software development company will allow vulnerable populations to benefit from the advantages of generative AI in the future.

Our study recruited people experiencing homelessness, one of the most underserved populations. The randomly invited study participants had a nonnegligible prior knowledge of AI technology’s existence, although they self-evaluated their knowledge as slightly below average. Previous research shows that people with lower socioeconomic status are slower to adopt new technology, and the rates of smartphone and internet use among people experiencing homelessness were lower than for those with similarly low socioeconomic status but more stable housing [14,50]. A 2023 international, multicenter, cross-sectional study assessing the attitudes of hospital patients toward AI in health care across 43 countries, including Hungary, found that patients have a predominantly favorable general view of AI in health care [51]. In Hungary, a representative survey published in September 2024 found that 79% of the population believed they knew what AI was, and 31% of respondents used chatbots and virtual customer service assistants [52].

In our study, participants’ attitudes toward the medical use of AI were slightly above average, meaning that they might be hesitant or neutral when it comes to trusting such services. This is in line with other Hungarian general populational findings [53]. In this survey, researchers asked respondents how they would feel if their family doctor or medical specialists would partly rely on AI during their care; overall, 41.2% of the respondents were neutral, 27.5% said they would feel rather bad or very bad, while 31.3% reported they would feel rather well or very well about it [53].

### Text Quality Evaluation

The ChatGPT-generated draft text variants had to be modified by the researchers as these versions contained a few severe grammatical errors in the Hungarian language; however, after such modifications were made, the texts were presentable and positively accepted by the focus groups. This might be due to generative AI software being most predominantly trained on Standard English texts, which means that in the case of small languages such as Hungarian, there is limited data available online for model training; therefore, large language models perform worse in such a “low resource” language compared to English or other “high resource” languages such as Spanish, Chinese, or Arabic [54].

This could partly explain why the assessment of the text variants by the SHeLL Editor showed such strong differences by readability, although the target group was defined in the prompts as people experiencing homelessness, implying generally lower health literacy levels. As Ayre et al [33] found when they were experimenting with prompt design, prompts that described specific health literacy principles (eg, simple language, active voice, minimal jargon) worked better with ChatGPT than prompts that described the target audience. This could suggest that social sector employees would greatly benefit from prompting skill enhancement concerning AI health-related text generation. The use of the official ChatGPT prompt engineering guide [55] or specific prompt design elements, such as in-context learning, could also aid the process [56].

Members of the focus groups generally stated that the various styles and tones might attract various subgroups and generations of people experiencing homelessness; however, they also noted that the motivational elements of text variants were stereotypical of a subgroup of people experiencing homelessness (rough sleepers) and lacking other prominent subgroups (eg, people accommodated in community shelters or temporary hostels). This could partly stem from the generalization bias challenge of LLM models such as the one behind ChatGPT. These models are trained on large datasets that may contain biases, stereotypes, and prejudiced language [49,57]. As a result, the model may unintentionally learn these biases and produce responses that are offensive or perpetuate harmful stereotypes, such as the one about people experiencing homelessness being represented as rough sleepers.

### Co-Design With Experts by Experience and Technology

In recent years, co-design, co-creation, co-production, or different forms of citizen engagement and collaboration of stakeholders have gained popularity in various fields, including social services for people experiencing homelessness [58]. The involvement of individuals with lived experience has also been shown to increase recruitment and follow-up rates in research projects, add to the validation of research findings, and generate more useful outputs [59,60]. This research project highlights these previous findings, as the involvement of the experts by experience group, as well as two focus groups, generated useful insights.

This experimental focus group study offered the opportunity to bring generative AI technology into the co-design process as a potential new element for the consideration of personnel working in the social or health sectors dealing with vulnerable subgroups, although the final benefits of this approach require further research and analysis. Our results showed that ChatGPT could produce usable material as a solid base for a health information material draft, which was acceptable for the target group, while the co-design process revealed additional benefits.

### Limitations

Our study had some limitations. As a qualitative study relying on focus groups and feedback questionnaires, the methods themselves posed certain drawbacks. Although focus groups encourage participation from vulnerable populations and do not rely on participant literacy, they offer a space where those individual perspectives that differ from the majority opinion might remain hidden due to overriding behavioral or cultural norms or a desire to be seen as conforming [61,62].

The study participants were selected from the urban homeless population in Budapest, Hungary, where socioeconomic conditions might differ from those in the countryside. In addition, participants represented people experiencing homelessness who had a connection to the social infrastructure; therefore, others not in touch with the Hungarian social service architecture were not represented in the study sample. For a qualitative study using focus groups and feedback questionnaires, the sample size was small, and this should be taken into account when drawing conclusions.

Regarding technology, the researchers used OpenAI’s most advanced freely accessible technology, ChatGPT 3.5, at the time of the research, while other generative AI software, such as Google’s Gemini (previously Bard), Claude, or Synthesia, were not used. The use of ChatGPT 3.5, or any other generative software for that matter, also raises the question of replicability; with the constant and rapid development of LLMs, it might become uncertain whether this research could be replicated with the same technological conditions. Regarding the text evaluation aspect, we did not use a baseline text variant produced by human hands, as we had the intention to involve only ChatGPT in the co-creation process, as well as to assess the quality of the text variants that emerged during the process.

### Conclusions

Our study revealed that health information materials generated by AI can be used by people experiencing homelessness in an oral cancer screening program. The co-design process revealed that the participants in the focus groups wanted to actively shape the drafts for the screening program and shared their ideas and insights on how to finalize the texts to avoid prevailing stereotypes about people experiencing homelessness and include more subgroups, as well as how to frame the text for various target audiences.

The group discussion also revealed some challenges of current LLM technology when using it without prior prompting experience. Based on our results, using the most up-to-date LLM technology, considering the health literacy and general language skills of vulnerable populations and avoiding generalization bias for this underrepresented group, and extensive prompt design upskilling of social workers and other groups of people aiming to produce health information material would be beneficial for future applications.

Moreover, via co-creation with members of the target audience, the final product might be more appealing to the target group of a health screening program. As a recommendation for its efficient use, offering prompt design training to personnel working in the social or health sectors may help maximize the impact of AI in client care.

## Supplementary material

10.2196/68316 Multimedia Appendix 1 ChatGPT prompts and responses.

10.2196/68316 Checklist 1 Consolidated Criteria for Reporting Qualitative Research checklist.

## Abbreviations

AI: artificial intelligence
COREQ: Consolidated Criteria for Reporting Qualitative Research
LLM: large language model
SHeLL: Sydney Health Literacy Lab
