# Supplementary material for: Co-Design of a Health Screening Program Fact Sheet by People Experiencing Homelessness and ChatGPT: Focus Group Study
Source: JMIR Form Res. 2025 Jul 4;9:e68316. doi: 10.2196/68316 (PMC12248257; doi:10.2196/68316)
Supplement: Multimedia Appendix 1 [file formative-v9-e68316-s001.docx]

**People Experiencing Homelessness and ChatGPT Co-Designed a Factsheet for a Health Screening Program: A Focus Group Study**

**Supplementary Material S02.**

Tool used for text generation: ChatGPT version 3.5.

**Prompt 1:** An oral cancer screening program is planned targeting homeless people. A patient information leaflet is needed to raise the level of participation in this vulnerable population. Please, provide a text aiming to inform homeless people about the importance of this initiative. The reasoning in the text should be based on scientific evidence regarding oral cancer. The text should be less than 150 words and in Hungarian language.

**Text Variant 1:**

Fontos informálni Önt az előtt, hogy az orális rákszűrő program fontos lehet az Ön számára. Tudományos kutatások szerint az utcán élők nagyobb esélyt hordoznak az orális rák kialakulására. Ennek oka lehet az egészségtelen életmód, a dohányzás, az alkoholfogyasztás és a táplálkozási hiányosságok. Az időben történő felismerés és kezelés kulcsfontosságú a betegség megelőzésében és kezelésében. A szájüregi rák korai stádiumban gyakran tünetmentes, ezért rendszeres szűrővizsgálatok segíthetnek az időben történő felismerésben. Kérjük, vegyen részt a programban, hogy segítsen megőrizni egészségét és jólétét.

**English version:**

It is important to inform you before an oral cancer screening programme may be important for you. Scientific research has shown that people living on the streets are more likely to develop oral cancer. This may be due to unhealthy lifestyles, smoking, alcohol consumption and nutritional deficiencies. Early detection and treatment is key to preventing and treating the disease. Oral cancer is often asymptomatic at an early stage, so regular screening can help to detect it early. Please get involved to help protect your health and well-being.

**Prompt 2:** An oral cancer screening program is planned targeting homeless people. A patient information leaflet is needed to raise the level of participation in this vulnerable population. Please, provide a text aiming to inform homeless people about the importance of this initiative. The reasoning in the text should be based on statistical evidence regarding oral cancer. The text should be less than 150 words and in Hungarian language.

**Text Variant 2:**

Kérjük, vegyen részt az orális rákszűrő programban, mert az utcán élők körében a szájüregi rák előfordulási aránya magasabb. Statisztikák szerint az egészségtelen életmód és a nehezen elérhető egészségügyi ellátás miatt az utcán élők nagyobb veszélyben vannak. Az időben történő szűrés lehetőséget ad a betegség korai felismerésére és kezelésére, növelve ezzel az esélyt a gyógyulásra. A részvétel segíthet megelőzni a súlyosabb problémákat és hozzájárulhat az Ön egészségének megőrzéséhez. Kérjük, ne hagyja ki ezt az lehetőséget!

**English version:**

Please take part in the oral cancer screening programme, as the incidence of oral cancer is higher among people living on the streets. Statistics show that people living on the streets are at higher risk due to unhealthy lifestyles and difficult access to healthcare. Timely screening allows for the detection and treatment of the disease early, increasing the chances of a cure. Getting involved can help prevent more serious problems and help keep you healthy. Please do not miss this opportunity!

**Prompt 3:** An oral cancer screening program is planned targeting homeless people. A patient information leaflet is needed to raise the level of participation in this vulnerable population. Please, provide a text aiming to inform homeless people about the importance of this initiative. The text should be as motivating as possible. The text should be less than 150 words and in Hungarian language.

**Text Variant 3:**

Ne hagyja ki az ingyenes orális rákszűrő programot! Az utcán élők körében az orális rák előfordulási aránya magasabb, de időben történő felismeréssel és kezeléssel megelőzhető! A részvétel segíthet az egészsége megőrzésében és az életminőség javításában. Ne engedje, hogy a helyzet korlátozza az egészségügyi ellátáshoz való hozzáférését! Egy rövid, ingyenes vizsgálat lehet az, ami megkönnyíti az esetleges problémák korai felismerését és kezelését. A gyógyulás esélyei sokkal nagyobbak, ha időben intézkedünk. Vegyen részt a programban, és tegyen az egészsége érdekében!

**English version:**

Don't miss the free oral cancer screening programme! The incidence of oral cancer is higher among people living on the streets, but it can be prevented with early detection and treatment. Participation can help you stay healthy and improve your quality of life. Don't let your situation limit your access to health care! A short, free check-up can be the key to helping you identify and treat any problems early. The chances of recovery are much higher if action is taken early. Get involved and take action for your health!

**Prompt 4:** An oral cancer screening program is planned targeting homeless people. A patient information leaflet is needed to raise the level of participation in this vulnerable population. Please, provide a text aiming to inform homeless people about the importance of this initiative. The text should be informal, familiar, using slang expressions. The text should be less than 150 words and in Hungarian language.

**Text Variant 4:**

Hé, srácok és csajok! Figyeljetek ide! Van egy jó hírem! Lesz egy ingyenes szájrák-szűrő program, ami kifejezetten rátok van szabva. Tudjátok, az utcán élők körében nagyobb az esélye a szájrák kialakulásának. De ne aggódjatok, mert most itt van ez a program, hogy segítsen nektek! Egy rövid vizsgálat, és máris sokat tehettek az egészségetekért. Tudjátok, minél hamarabb derül ki egy ilyen dolog, annál könnyebb rajta segíteni. Szóval ne habozzatok, gyertek el és vegyetek részt! Egyszerű, ingyenes, és sokat segíthet nektek! Ne hagyjátok ki, srácok, fontos dologról van szó!

**English Version:**

Hey, guys! Listen up! I have good news! There will be a free oral cancer screening program specially for you. You see, people living on the streets are more likely to develop oral cancer. But don't worry, because now this program is here to help you! A quick check-up and you can do a lot for your health. You know that the sooner you find out, the easier it is to help. So don't hesitate, come and get involved! It's simple, free and can help you a lot! Don't miss it, guys, it's important!

**Prompt 5:** An oral cancer screening program is planned targeting homeless people. A patient information leaflet is needed to raise the level of participation in this vulnerable population. Please, provide a text aiming to inform homeless people about the importance of this initiative. The text should be formatted as a clickbait news article. The text should be less than 150 words and in Hungarian language.

**Text Variant 5:**

"FIGYELEM! INGYENES SZÁJRÁK-SZŰRÉS AZ UTCÁN ÉLŐKNEK! NE HAGYD KI!

Szájrák-riadó az utcán élőknek! Tudtad, hogy az utcán élők körében nagyobb az esélye a szájrák kialakulásának? De most itt a megoldás! Egy új, ingyenes szájrák-szűrő program érkezik, hogy megvédjen téged és szeretteidet! Ne veszítsd el az esélyt a korai felismerésre és a gyógyulásra! Csak egy rövid vizsgálat, és már megtettél egy nagy lépést az egészségedért! Kattints és regisztrálj most! Ne hagyd ki ezt a lehetőséget, mert az egészséged a tét! Megéri!"

**English Version:**

"WARNING! FREE ORAL CANCER SCREENING FOR PEOPLE LIVING ON THE STREETS! DON'T MISS IT!

Oral cancer screening for people living on the streets! Did you know that people living on the streets are more likely to develop oral cancer? But now here's the solution! A new free oral cancer screening program is coming to protect you and your loved ones! Don't miss your chance for early detection and a cure! With just a short exam, you've taken a big step for your health! Click and register now! Don't miss this opportunity because your health is at stake! It's worth it!"

**Prompt 6:** An oral cancer screening program is planned targeting homeless people. A patient information leaflet is needed to raise the level of participation in this vulnerable population. Please, provide a text aiming to inform homeless people about the importance of this initiative. The text should be structured in a bullet-point format. The text should be less than 150 words and in Hungarian language.

**Text Variant 6:**

- Ingyenes szájrák-szűrő program az utcán élőknek!
- Az utcán élők körében magasabb az orális rák kockázata.
- Időben történő felismeréssel és kezeléssel megelőzhető a súlyosabb problémák kialakulása.
- A szűrővizsgálat segíthet az esetleges rákos elváltozások korai felfedezésében.
- Rövid és ingyenes vizsgálat, melynek során szakemberek segítenek Önnek az egészsége megőrzésében.
- Vegyen részt a programban, hogy hozzájáruljon saját egészségének védelméhez és a betegség korai felismeréséhez.

**English Version:**

- Free oral cancer screening programme for people living on the streets!
- People living on the streets have a higher risk of oral cancer.
- Early detection and treatment can prevent more serious problems from developing.
- Screening can help detect possible cancers early.
- It's a short and free test, with professionals to help you stay healthy.
- Take part to help protect your health and detect disease early.
