# Supplementary material for: Co-Design of a Health Screening Program Fact Sheet by People Experiencing Homelessness and ChatGPT: Focus Group Study
Source: JMIR Form Res. 2025 Jul 4;9:e68316. doi: 10.2196/68316 (PMC12248257; doi:10.2196/68316)
Supplement: Checklist 1 [file formative-v9-e68316-s002.docx]

**Supplementary Material S01.**

**Completed checklist of the consolidated criteria for reporting qualitative research (COREQ)^1^**

| **No. Item** | **Description** |
| --- | --- |
| **Domain 1: Research team and reﬂexivity** |  |
| *Personal Characteristics* |  |
| 1. Facilitator of Focus Groups | Focus Groups were conducted by a moderator professional in the field of research, while another researcher took up the role of observer in one focus group |
| 2. Credentials | MD, PhD |
| 3. Occupation | Research Director, Physician and Sociologist |
| 4. Gender | Male, Female |
| 5. Experience and training | Several years of experience in field work |
| *Relationship with participants* |  |
| 6. Relationship established | Participants knew the moderator, which created the atmosphere of trust, which is very important for this specific population |
| 7. Participant knowledge of the interviewer | Participants were informed in oral form in advance of the purpose of the focus groups |
| 8. Interviewer characteristics | Interests in the research topics |
| **Domain 2: study design** |  |
| *Theoretical framework* |  |
| 9. Methodological orientation and Theory | Text analysis in a co-design process |
| *Participant selection* |  |
| 10. Sampling | Convenience sampling |
| 11. Method of approach | Face-to-face conversations in 3 focus groups, participants were approached personally in 3 shelters by social workers with the option to participate |
| 12. Sample size | 23 participants in 3 focus groups |
| 13. Non-participation | 3 people decided not to participate: 2 persons due to scheduling problems and 1 person due to the difficulty of the topic |
| *Setting* |  |
| 14. Setting of data collection | Shelters |
| 15. Presence of non-participants | Only researchers (moderator and observer) and participants of the focus groups were present |
| 16. Description of sample | Participants represented homeless shelter residents of 3 homeless shelters in Budapest operated by the Hungarian Charity Service of the Order of Malta (Miklós Street Integrated Homeless Care Center, Homeless Care Center at Bem Rakpart, Galvani Street Homeless Care Center)  3 focus groups were organized (N=6;N=10;N=7), 2 ad hoc groups and 1 group were participants represented the members of an experts by experience group  In terms of demographic composition, altogether 3 females and 20 males participated, and the mean ages for the focus groups were the following: 55.83 years; 61.5 years; 53.57 years. They were all people experiencing homelessness living in Budapest, Hungary. |
| *Data collection* |  |
| 17. Interview guide | Focus groups were centered around the textual analysis of factsheet variants previously generated by ChatGPT, and questionnaires about artificial intelligence |
| 18. Repeat interviews | N/A |
| 19. Audio/visual recording | Audio recordings |
| 20. Field notes | Field notes at the setting; ex-post notes about the circumstances of the focus groups |
| 21. Duration | For all 3 focus groups, discussions lasted 40-55 minutes |
| 22. Data saturation | No |
| 23. Transcripts returned | No |
| **Domain 3: analysis and ﬁndings** |  |
| *Data analysis* |  |
| 24. Number of data coders | 2 |
| 25. Description of the coding tree | N/A |
| 26. Derivation of themes | Textual analysis resulted in comparison of AI generated text variants based on their strengths and weaknesses, as well as their readability level based on the text variants’ grade reading score and text complexity. |
| 27. Software | N/A |
| 28. Participant checking | No |
| *Reporting* |  |
| 29. Quotations presented | Participant quotations are presented to illustrate the ﬁndings; each quotation is de-identified. |
| 30. Data and ﬁndings consistent | Yes |
| 31. Clarity of major themes | N/A |
| 32. Clarity of minor themes | N/A |

1. *Tong A, Sainsbury P, Craig J. Consolidated criteria for reporting qualitative research (COREQ): a 32-item checklist for interviews and focus groups. International Journal for Quality in Health Care. 2007. Volume 19, Number 6: pp. 349 – 357.*
